# Time-dependent effect of GLP-1 receptor agonists on cardiovascular benefits: a real-world study

**DOI:** 10.1186/s12933-023-01800-z

**Published:** 2023-03-25

**Authors:** Sara Piccini, Giuseppe Favacchio, Cristina Panico, Emanuela Morenghi, Franco Folli, Gherardo Mazziotti, Andrea Gerardo Lania, Marco Mirani

**Affiliations:** 1 Department of Biomedical Sciences, Humanitas University, Via Rita Levi Montalcini 4, 20072 Pieve Emanuele, Milan Italy; 2 Endocrinology, Diabetology and Andrology Unit, IRCCS Humanitas Research Hospital, Via Manzoni 56, 20089 Rozzano, Milan Italy; 3 Department of Cardiology, IRCCS Humanitas Research Hospital, Rozzano, Milan Italy; 4 Biostatistic Unit, IRCCS Humanitas Research Hospital, Via Manzoni 56, 20089 Rozzano, Milan Italy; 5 Endocrinology and Metabolism, Department of Health Science, Università Degli Studi di Milano, Milan, Italy

**Keywords:** Diabetes, GLP-1 receptor agonists, Cardiovascular events, Real-world

## Abstract

**Background:**

Glucagon-like peptide-1 receptor agonists (GLP-1 RAs) have shown cardiovascular benefits in cardiovascular outcome trials in type 2 diabetes mellitus. However, the most convincing evidence was obtained in subjects with established cardiovascular (CV) disease. We analyzed the determinants of GLP-1 RA-mediated CV protection in a real-world population of persons with type 2 diabetes with and without a history of CV events with long-term follow-up.

**Methods:**

Retrospective cohort study of 550 individuals with type 2 diabetes (395 in primary CV prevention, 155 in secondary CV prevention), followed at a single center after the first prescription of a GLP-1 RA between 2009 and 2019. CV and metabolic outcomes were assessed.

**Results:**

Median duration of follow-up was 5.0 years (0.25–10.8) in primary prevention and 3.6 years (0–10.3) in secondary prevention, with a median duration of treatment of 3.2 years (0–10.8) and 2.5 years (0–10.3) respectively. In the multivariable Cox regression model considering GLP-1 RA treatment as a time-dependent covariate, in the primary prevention group, changes in BMI and glycated hemoglobin did not have an impact on MACE risk, while age at the time of GLP-1 initiation (HR 1.08, 95% CI 1.03–1.14, *p* = 0.001) and GLP-1 RA cessation by time (HR 3.40, 95% CI 1.82–6.32, *p* < 0.001) increased the risk of MACE. Regarding the secondary prevention group, only GLP-1 RA cessation by time (HR 2.71, 95% CI 1.46–5.01, *p* = 0.002) increased the risk of MACE. With respect to those who withdrew treatment, subjects who continued the GLP-1 RA had significantly greater weight loss and lower glycated hemoglobin levels during follow-up.

**Conclusions:**

In this real-world type 2 diabetes population, discontinuation of GLP-1 RA treatment was associated to a higher risk of major cardiovascular events, in both subjects with and without a history of CV events.

## Background

GLP-1 receptor agonists (GLP-1 RAs) have been employed for over a decade for the treatment of type 2 diabetes mellitus. At present, several GLP-1 RA formulations are available: liraglutide and lixisenatide are subcutaneously administered once per day; dulaglutide, semaglutide, exenatide LAR are injected weekly. GLP-1 RAs share multiple mechanisms of action, including improvement of insulin secretion in response to hyperglycemia, suppression of glucagon hypersecretion, deceleration of gastric emptying reducing post-meal glycemic excursions, and changes in appetite and satiety leading to a reduction in body weight [1–4].

As for all new anti-diabetic medications, before approval for clinical use, GLP-1 RAs demonstrated cardiovascular safety in placebo controlled cardiovascular outcome trials (CVOTs). The primary endpoint of most of these studies were major adverse cardiovascular events (MACE), a combined endpoint of either cardiovascular (CV) death or non-fatal myocardial infarction or stroke. All GLP-1 RA, except for lixisenatide, showed decreased incidence of MACE, which was statistically significant in four of the studies (LEADER for liraglutide, SUSTAIN-6 for weekly semaglutide, Harmony Outcomes for albiglutide and REWIND for dulaglutide) [5–11].

GLP-1 RA CVOTs were quite heterogeneous in study design, sample size, duration, and proportion of patients with established CV disease (CVD). The individual studies were not powered to evaluate individual CV events, and, in fact, single CV events were seldom significantly decreased (for instance, liraglutide significantly decreased CV and all-cause mortality in the LEADER trial). Nevertheless, several meta-analyses of CVOTs showed that GLP-1 RA decreased MACE risk by 14–16%, and, as a class, they did significantly decrease hazard ratios for individual events, including death from CV causes, fatal or non-fatal stroke, fatal or non-fatal myocardial infarction, and all-cause mortality [12–15]. Interestingly, two of the most recent meta-analyses that included subgroup analyses of subjects with or without established CVD, suggest similar favorable effects in patients with and without CVD [12, 14]. However, the number of events in primary prevention was small, due to the relatively short duration of the trials and the lower risk in these subjects.

GLP-1 RAs exert their beneficial CV effects through incompletely characterized mechanisms. Between the potential determinants of CV benefits, GLP-1 RAs modify CV risk factors by reducing body weight, systolic blood pressure, plasma LDL cholesterol and tryglycerides and by improving glycemic control (reduced HbA1c, avoidance of severe hypoglycemia). In addition, GLP-1 RAs have been shown to exert direct effects on the CV system, potentially leading to improved endothelial function, improved cardiac function under conditions of coronary ischemia, anti-inflammatory, and anti-atherosclerotic effects [16].

The first GLP-1 RA to be approved in Italy was exenatide b.i.d. in 2008, and since then we have collected more than 10 years of experience on diabetic patients treated with these agents. Considering the difference between randomized controlled trials (RCT) and real-life clinical practice, we analyzed the determinants of GLP-1 RA-mediated CV protection in a real-world population of patients with type 2 diabetes attending the outpatient clinic of a large university hospital located in the metropolitan area of Milan, Italy.

## Methods

This was a retrospective cohort study of patients with type 2 diabetes mellitus who were followed at a single high-volume center (Humanitas Research Hospital, Milan). The institutional Ethical Committee approved this study and patients gave standard written informed consent to use their anonymized clinical data for research purposes.

Using electronic medical records, patients receiving for the first time an injectable GLP-1 receptor agonist (i.e.: liraglutide, exenatide, lixisenatide, dulaglutide, semaglutide) between December 1st, 2009, and December 31st, 2019, were identified. The date of the visit in which the GLP-1 RA was first prescribed, was considered as baseline. Follow-up data until December 31st, 2021, were included. Inclusion criteria were the following: age > 18 years, signed informed consent, a diagnosis of type 2 diabetes mellitus, being naïve to GLP-1 RA, and having a minimum follow-up of 2 years at our center with complete availability of the clinical and biochemical data relating to weight, glycemic control and CV events. We excluded patients who started a GLP-1 RA after 2020, since they started treatment during the COVID-19 pandemic and were less likely to have reliable follow-up data. Exclusion criteria were the following: malignant tumor first diagnosed, relapsed, or undergoing active treatment (i.e.: chemotherapy, targeted therapies, radiotherapy) at the time of the first prescription of a GLP-1 RA; gestational diabetes and any form of secondary diabetes (e.g.: diagnosis of active Cushing's disease, pancreatectomy, corticosteroid treatment); bariatric surgery at any time during follow-up; diagnosis of type 1 diabetes or latent autoimmune diabetes of the adult (LADA).

Treatment persistence, side effects, cardiovascular and metabolic outcomes were evaluated during the follow-up period.

The dataset supporting the conclusions of this article is available in the Zenodo repository, https://doi.org/10.5281/zenodo.7533472.

### Clinical data

Demographic (age and gender) and clinical characteristics were evaluated at baseline (first prescription of a GLP-1 RA). Weight and height were measured and used to calculate body mass index (BMI). The presence of diabetes complications (retinopathy, nephropathy, neuropathy) and of additional CV risk factors was assessed (arterial hypertension, dyslipidemia, obesity, smoking, coronary, carotid, or lower extremity artery stenosis > 50%, left ventricular hypertrophy). Arterial hypertension was defined as a systolic blood pressure (BP) ≥ 140 mmHg or a diastolic BP ≥ 90 mmHg or current antihypertensive treatment. Dyslipidemia was defined by elevated serum lipid levels according to the current guidelines at the time of the baseline visit or current lipid-lowering treatment. Established CVD was defined as previous stroke, MI, unstable angina, myocardial ischemia on imaging or stress test, or coronary, carotid, or peripheral artery revascularization.

Medication history included glucose-lowering treatments used until the baseline visit, use of cardioactive drugs, including statins, ezetimibe, proprotein convertase subtilisin/kexin type 9 (PCSK-9) inhibitors, antiplatelet drugs, antihypertensive drugs, angiotensin-converting enzyme (ACE) inhibitors, angiotensin receptor blockers (ARB), mineralocorticoid receptor antagonists (MRA), and sacubitril/valsartan. All glucose-lowering therapies prescribed during the follow-up period were recorded. Duration of treatment with GLP-1 RA was computed, and, in case of discontinuation, the reason was evaluated. During each visit, self-reported treatment adherence to glucose-lowering medication was assessed. Duration of treatment with GLP-1 RA was computed, and, in case of discontinuation, the reason was evaluated. Treatment discontinuation was defined as the interruption of GLP-1 RA therapy for at least 3 months. If a subject resumed a GLP-1 RA after 3 months, his follow up ended at that time.

Laboratory data and body weight were recorded at baseline, and at 1 year, 2, 4, 6, 8 and 10 years (when available), and at the end of follow-up (the most recent visit available for each patient).

The primary outcome was a composite of major adverse CV events (MACE): non-fatal myocardial infarction (MI) or unstable angina, non-fatal stroke, and all-cause death. All-cause death was identified from the administrative data repository of the Lombardy Region (Italy). Death from any cause was used in place of cardiovascular death, due to uncertainty in establishing the latter in most of the cases.

Glycemic control and weight changes over time were also assessed.

### Statistical analysis

Due to the exploratory nature of the study, we enrolled all the patients receiving any GLP-1 receptor agonist in a 10-year period, estimating the number of subjects to be around 500. Due to the deep expected difference in MACE incidence, we conducted our analysis in parallel in patients with or without a previous CV event.

Data were described as number and percentage, if categorical, or mean and standard deviation, if continuous. If required by the description, a 95% confidence interval has been added. Adherence to Gaussian distribution was verified with the Shapiro–Wilk test.

Survival from MACE was explored with survival analysis, considering as time to failure the first occurrence of non-fatal myocardial infarction (MI) or unstable angina, non-fatal stroke, and all-cause death, or the last contact date on censored patients. All potential prognostic factors were submitted to univariable proportional hazard Cox regression analysis. To include the duration of treatment in the analysis, we used a Cox regression model considering GLP-1 RA treatment as a time dependent covariate. Each patient with MACE was matched with a censored patient by year of therapy initiation, corrected for age at the beginning of therapy, BMI and glycated hemoglobin considered at beginning and end of therapy.

Changes in biological parameters (weight, blood glucose, and glycated hemoglobin) were described, comparing persistent and non-persistent GLP-1 users. Difference between the two groups were explored with Mann Whitney test, due to the non-Gaussian nature of the variables.

A p under 0.05 was considered as significant. All analyses were performed with Stata version 17.

## Results

### Study population

**Fig. 1:**
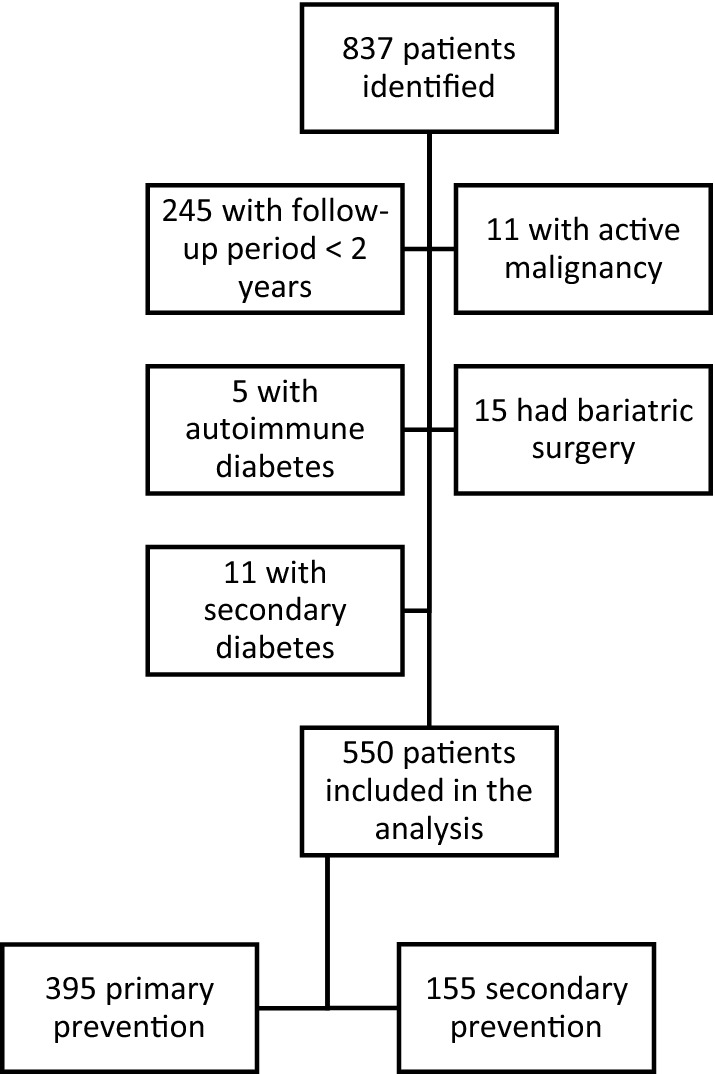
Study profile

A total of 837 patients had a first prescription for a GLP-1 RA during the index period. 550 patients met the criteria for inclusion (65.7%). Of those not included, 245 patients (29.3%) did not have the minimum follow-up period of 2 years, 11 (1.3%) had a concomitant active malignancy, 5 (0.6%) were classified as LADA, 15 (1.8%) had bariatric surgery during follow-up, and 11 (1.3%) had secondary diabetes (Fig. 1).

Subjects were split into two groups, namely a primary prevention group of persons without a history of CV events (N = 395 subjects), and a secondary prevention group of patients with a history of CV events (N = 155 subjects), defined as previous stroke, MI, unstable angina, myocardial ischemia on imaging or stress test, or coronary, carotid, or peripheral revascularization.

Baseline clinical and laboratory characteristics of study participants are shown in Table 1. All patients were residents in the Milan metropolitan area.

**Table 1:** Baseline characteristics

	Primary prevention	Secondary prevention
N	395	155
Gender (males)	193 (48.86%)	119 (76.77%)
Age, years	61.6 ± 10.1	66.3 ± 7.8
BMI, kg/m^2^	34.2 ± 5.8	32.2 ± 5.9
BMI < 30	92 (23.35%)	62 (40.00%)
BMI 30–35	148 (37.56%)	56 (36.13%)
BMI > 35	154 (39.09%)	37 (23.87%)
Obese (BMI > 30 kg/m^2^)	302 (76.5%)	93 (60.0%)
Diabetes duration, years	8 (0–48)	11 (0–50)
Diabetes duration > 10 years	136 (34.43%)	78 (50.32%)
sBP	137.9 ± 15.6	132.9 ± 14.7
dBP	79.9 ± 8.8	76.6 ± 8.3
Fasting blood glucose, mg/dL	174.1 ± 53.8	161.9 ± 50.2
HbA1c, %	8.22 ± 1.36	7.87 ± 1.33
HbA1c < 7%	50 (12.66%)	35 (22.58%)
Total cholesterol, mg/dL	181.6 ± 38.6	149.3 ± 32.8
HDL, mg/dL	45.8 ± 11.3	41.8 ± 10.3
Tryglicerides, mg/dL	178.4 ± 122.4	159.4 ± 79.6
LDLc^a^, mg/dL	102.7 ± 34.1	76.3 ± 26.6
Creatinine, mg/dL	0.86 ± 0.26	1.01 ± 0.32
eGFR, ml/min/1.73 m^2^	85.3 ± 19.3	76.7 ± 20.6
Proteinuria	11 (2.89%)	9 (6.04%)
Diabetic kidney disease	91 (23.27%)	52 (33.77%)
Diabetic retinopathy	51 (12.9%)	37 (23.9%)
Smoking status		
Never	236 (59.75%)	75 (48.39%)
Current smokers	71 (17.97%)	22 (14.19%)
Former smokers	88 (22.28%)	58 (37.42%)
Ischemic heart disease	0	137 (88.39%)
Stroke	0	8 (5.16%)
Peripheral artery revascularization	0	28 (18.06%)
Arterial hypertension	296 (74.94%)	150 (96.77%)
Dyslipidemia	247 (62.53%)	149 (96.13%)
> 50% carotid, coronary, or peripheral artery stenosis	22 (5.6%)	31 (20.6%)
Heart failure	7 (1.8%)	13 (8.4%)

LDLc was calculated using the Friedewald formula. eGFR, estimated glomerular filtration rate calculated with the CKD-EPI formula. sBP, dBP, systolic and diastolic blood pressure, respectively

^a^Missing values in 68 subjects in primary prevention and in 27 subjects in secondary prevention

In the primary prevention group, mean age was 61.6 years, 48.9% were males, mean diabetes duration was 8 years, mean glycated hemoglobin at baseline was 8.2%, and 12.7% had a glycated hemoglobin below 7%. The mean duration of follow-up was 5 years. In the secondary prevention group, mean age was 66.3 years, 76.8% were males, mean diabetes duration was 11 years, mean glycated hemoglobin at baseline was 7.8%, and 22.6% had a glycated hemoglobin below 7%.

At baseline, mean BMI was 34.2 and 32.2 kg/m^2^ in the primary prevention and in the secondary prevention groups respectively. In those without a history of CV events, 62.5% had dyslipidemia, 74.9% had arterial hypertension, 76.5% were obese (BMI > 30 kg/m^2^), 18.0% were active smokers, 22.3% were former smokers, and 1.8% had a diagnosis of heart failure. In the secondary prevention group, 88.4% had a history of ischemic heart disease, 5.2% had had a stroke, and 18.1% had received an arterial revascularization procedure, 60% were obese (BMI > 30 kg/m^2^), 14.2% were active smokers, 37.4% were former smokers, and 8.4% had a diagnosis of heart failure.

### Treatment patterns

Before the baseline visit, most patients were taking metformin (86.8% in primary prevention, 83.2% in secondary prevention), whereas 36.7% in primary prevention and 32.3% in secondary prevention were treated with either sulfonylureas or meglitinides, 21% were on insulin in the primary prevention group and 27.7% in the secondary prevention group, 23.3% and 21.3% respectively were on DPP-4 inhibitors, while very few were taking SGLT2 inhibitors (Table 2).

**Table 2:** Treatment patterns at baseline

	Primary prevention	Secondary prevention
*Ongoing anti-diabetic treatments before baseline visit*		
Metformin	343 (86.8%)	129 (83.2%)
Pioglitazone	68 (17.2%)	23 (14.8%)
Acarbose	5 (1.3%)	2 (1.3%)
Sulfonylureas or meglitinides	145 (36.7%)	50 (32.3%)
Insulin	83 (21%)	43 (27.7%)
SGLT2 inhibitors	12 (3.0%)	5 (3.2%)
DPP4 inhibitors	92 (23.3%)	33 (21.3%)
*Cardioactive therapies at baseline*		
Statins	177 (44.8%)	140 (90.3%)
Ezetimibe	17 (4.3%)	19 (12.3%)
PCSK-9 inhibitors	0	1 (0.6%)
Antiplatelet agents	113 (28.6%)	147 (94.8%)
Anti-hypertensives	282 (71.4%)	151 (97.4%)
ACEi o ARBs o MRAs	244 (61.8%)	132 (85.2%)
*GLP-1 RA prescribed at baseline*		
Lixisenatide	1 (0.3%)	0
Exenatide BID	6 (1.5%)	1 (0.6%)
Exenatide LAR	4 (1.0%)	0
Liraglutide	247 (62.5%)	92 (59.4%)
Dulaglutide	134 (33.9%)	62 (40%)
Semaglutide (weekly)	3 (0.8%)	0

SGLT2, sodium-glucose cotransporter-2; DPP4, Dipeptidyl peptidase-4; PCSK-9, Proprotein convertase subtilisin/kexin type 9; ACEi, angiotensin-converting enzyme inhibitor; ARB, angiotensin II receptor blocker; MRA, mineralocorticoid receptor antagonist

As shown in Table 2, in the primary prevention group, 71.4% of the subjects were treated with anti-hypertensive drugs, 28.6% were on anti-platelet drugs, while statins and ezetimibe were used by 44.8% and 4.3% of the subjects respectively. At baseline, 14 of the subjects with hypertension and 61 of those with dyslipidemia were newly diagnosed, and therefore they were not yet receiving pharmacological treatments. In those with previous CV events, nearly all patients were receiving cardioactive therapies in secondary prevention.

The mean duration of follow-up was 3.6 years. After initiating the GLP-1 RA, median duration of treatment was 3.2 years in primary prevention and 2.5 years in secondary prevention (Table 3).

**Table 3:** Treatment patterns during follow-up

	Primary prevention	Secondary prevention
N	395	155
Duration of follow-up	5.0 (0.25–10.8)	3.6 (0–10.3)
*GLP-1 RA during follow-up*^a^		
Lixisenatide	2 (0.5%)	0
Exenatide BID	6 (1.5%)	1 (0.6%)
Exenatide LAR	12 (3.0%)	3 (1.9%)
Liraglutide	257 (65.1%)	94 (60.6%)
Dulaglutide	182 (46.1%)	80 (51.6%)
Semaglutide (weekly)	63 (15.9%)	7 (4.5%)
Insulin degludec + liraglutide	21 (5.3%)	5 (3.2%)
*Duration of GLP-1 treatment, years*	3.2 (0–10.8)	2.5 (0–10.3)
GLP-1 RA discontinuation	159 (40.25%)	63 (40.65%)
*Reasons for GLP-1 RA discontinuation*		
GI symptoms	50 (31.4%^b^)	24 (38.1%^c^)
Inefficacy	74 (46.5%^b^)	25 (40.0%^c^)
Noncompliance	11 (6.9%^b^)	3 (4.8%^c^)
Other (e.g.: drug discontinued during hospitalizations, prescription expired, switch to SGLT2i…)	11 (6.9%^b^)	3 (4.8%^c^)
Worsening of kidney function	5 (3.1%^b^)	1 (1.6%^c^)
Malaise, fatigue, dizziness or myalgias	4 (2.5%^b^)	2 (3.2%^c^)
Allergic or cutaneous reactions	3 (1.9%^b^)	2 (3.2%^c^)
Incident pancreatitis or biliary disorders	2 (1.3%^b^)	1 (1.6%^c^)
Tachycardia	1 (0.6%^b^)	1 (1.6%^c^)
*Treatment persistence*		
GLP-1 RA ongoing at year 1	333 (84.3%)	121 (78.1%)
GLP-1 RA ongoing at year 2	297 (75.2%)	104 (67.1%)
GLP-1 RA ongoing at year 4	163 (64.9%^d^)	42 (56%^d^)
GLP-1 RA ongoing at year 6	100 (57.1%^d^)	24 (55.8%^d^)
GLP-1 RA ongoing at year 8	65 (60.2%^d^)	14 (50.0%^d^)
GLP-1 RA ongoing at year 10	15 (53.6%^d^)	3 (75%^d^)
*Switched to a different GLP-1 RA*	133 (33.7%)	32 (20.6%)
Treated with a single molecule	262 (66.3%)	123 (79.4%)
Switched to lixisenatide	0	0
Switched to exenatide BID	0	0
Switched to exenatide LAR	6 (1.5%)	3 (1.9%)
Switched to liraglutide	9 (2.3%)	2 (1.3%)
Switched to dulaglutide	46 (11.6%)	17 (11.0%)
Switched to semaglutide	56 (14.2%)	6 (3.9%)
Switched to insulin degludec + liraglutide	15 (3.8%)	4 (2.6%)
*Anti-diabetic medications taken with GLP-1 RAs*		
Metformin	359 (90.89%)	134 (86.45%)
Pioglitazone	86 (21.77%)	15 (9.68%)
Acarbose	9 (2.28%)	1 (0.65%)
Sulfonylureas/meglitinides	158 (40.00%)	53 (34.19%)
Insulin	109 (27.59%)	42 (27.10%)
SGLT-2 inhibitors	13 (3.29%)	4 (2.58%)
*Anti-diabetic medications after GLP-1 RA withdrawal*	N = 159	N = 63
Metformin	139 (86.3%^b^)	51 (82.3%^c^)
Pioglitazone	32 (19.9%^b^)	5 (8.1%^c^)
Acarbose	8 (5.0%^b^)	2 (3.2%^c^)
Sulfonylureas/meglitinides	84 (52.2%^b^)	26 (41.9%^c^)
Insulin	100 (62.1%^b^)	43 (69.4%^c^)
SGLT-2 inhibitors	70 (44.03%^b^)	25 (39.68%^c^)
DPP-4 inhibitors	40 (24.8%^b^)	22 (35.5%^c^)
*Severe hypoglycemic events*	5 (1.0%; 3 patients with 1 episode, 1 patient with 2 episodes)	0

^a^During follow-up, patients could switch to different GLP-1 RAs from the ones prescribed at baseline

^b^Percentage of those who discontinued the drug, n = 159 in primary prevention

^c^Percentage of those who discontinued the drug, n = 63 in secondary prevention

^d^The percentages refer to the numbers of patients still on follow-up: year 4, primary prevention 251, secondary prevention 75; year 6 primary 175, secondary 43; year 8 primary 108, secondary 28; year 10 primary 28, secondary 4

Liraglutide and dulaglutide were the most frequently prescribed GLP-1 RAs (liraglutide: 65.1% and 60.6%, dulaglutide: 46.1% and 51.6% in primary and in secondary prevention respectively). 33.7% of the subjects in primary prevention and 20.6% of those in secondary prevention were prescribed two or more different medications during follow-up, mostly because of switching from daily to weekly formulations.

The majority of the subjects in both groups were on metformin in association to the GLP-1 RA, more than one third was on sulfonylureas or meglitinides, about 27% were on insulin, while pioglitazone was used by 21.8% of those in primary prevention and by 9.7% of those with a history of CV events (Table 3).

The percentage of patients still taking the GLP-1 RA gradually declined during follow-up, from 84% in primary prevention and 78% in secondary prevention at one year, to 65% and 56% at four years, and 60% and 50% at eight years, respectively (Table 3). The most common causes of drug discontinuation were either gastrointestinal side effects (31% of those who discontinued the GLP-1 in primary prevention and 38% in secondary prevention) or inefficacy to achieve glycemic targets or weight loss (46% in primary prevention and 40% in secondary prevention). With respect to glycemic control, target HbA1c was personalized according to the applicable guidelines at the time of the visit. Clinically relevant weight change was defined as a weight loss of at least 5%.

After GLP-1 discontinuation, half of the patients started a sodium-glucose cotransporter-2 (SGLT-2) inhibitor (48% in primary prevention and 58% in secondary prevention), whereas the proportion of insulin and sulfonylurea users increased (insulin: 62% and 69%, sulfonylureas/meglitinides: 52% and 42% in primary and in secondary prevention respectively). 25% of those in primary prevention and 36% of those in secondary prevention were switched to a DPP-4 inhibitor (Table 3).

### CV outcomes

During a median follow-up of 5.0 years in primary prevention and 3.6 years in secondary prevention, the primary composite outcome (MACE) occurred in 34 patients (8.6%) in the first group and 32 patients (20.7%) in latter. The total number of events in primary prevention was: 9 (2.28%) myocardial infarctions or unstable anginas, 6 (1.52%) strokes, and 19 (4.81%) deaths. In those with a history of previous CV events, there were 11 (7.10%) myocardial infarctions or unstable anginas, 6 (3.87%) strokes, and 15 (9.68%) deaths. Data are summarized in Table 4.

**Table 4:** CV events during follow-up

	Primary prevention	Secondary prevention
n	395	155
*MACE*	34 (8.61%)	32 (20.65%)
Stroke	6 (1.52%)	6 (3.87%)
Myocardial infarction or unstable angina	9 (2.28%)	11 (7.10%)
All-cause death	19 (4.81%)	15 (9.68%)

The main composite CV outcome (MACE) included non-fatal myocardial infarction or unstable angina, non-fatal stroke, all-cause death

In the primary prevention group, after GLP-1 RA withdrawal, patients who experienced a MACE, compared to patients without events were taking less frequently metformin (59% vs. 92%, *p* = 0.001), pioglitazone (0% vs. 22%, *p* = 0.042) and SGLT-2i (17.65% vs. 50%, *p* = 0.018), while insulin users were more numerous (88% vs. 60%, *p* = 0.031). In the secondary prevention group, fewer patients who had a MACE were using SGLT-2i (14.29% vs. 53%, *p* = 0.013), while more patients were on insulin (93% vs. 63%, *p* = 0.044). Data are summarized in Table 5.

**Table 5:** Diabetes medications after GLP-1 RA withdrawal

N	Primary prevention			Secondary prevention		
	MACE	No MACE	*p*	MACE	No MACE	*P*
	17	135		14	43	
Metformin	10 (58.82%)	124 (91.85%)	0.001	9 (64.29%)	37 (86.05%)	0.115
Pioglitazone	0	30 (22.22%)	0.042	0	4 (9.30%)	0.563
Acarbose	1 (5.88%)	6 (4.44%)	0.551	1 (7.14%)	1 (2.33%)	0.434
Sulfonylureas/meglitinides	8 (47.06%)	78 (57.78%)	0.552	6 (42.86%)	17 (40.48%)	0.875
Insulin	15/17 (88.24%)	81 (60.00%)	0.031	13 (92.86%)	27 (62.79%)	0.044
SGLT-2 inhibitors	3 (17.65%)	67 (49.63%)	0.018	2 (14.29%)	23 (53.49%)	0.013
DPP-4 inhibitors	2 (11.76%)	37 (27.82%)	0.240	5 (35.71%)	17 (39.53%)	0.705

In the univariable Cox regression analysis for those in primary prevention (Table 6) older age (HR 1.10, 95% CI 1.04–1.15, *p* < 0.001), longer diabetes duration (HR 1.07, 95% CI 1.03–1.10, *p* < 0.001), the presence of kidney disease (HR 2.17, 95% CI 1.10–4.31, *p* = 0.026), were associated to an increased HR for MACE, diastolic blood pressure at baseline (HR 0.96, 95% CI 0.93–1.00, *p* = 0.046), and better kidney function at baseline (HR 0.97, 95% CI 0.96–0.99, *p* < 0.001) were associated to a lower HR for MACE.

**Table 6:** Results of the univariable Cox regression analysis

Univariable analysis	Primary prevention		Secondary prevention	
	HR (95% CI)	*p*	HR (95% CI)	*p*
Gender (male)	0.91 (0.46–1.81)	0.780	1.17 (0.52–2.65)	0.698
Age, years	1.10 (1.04–1.15)	< 0.001	1.05 (1.00–1.11)	0.038
*Smoking*				
Never	1		1	
Current	0.63 (0.21–1.82)	0.391	1.09 (0.40–3.01)	0.864
Former	0.90 (0.39–2.10)	0.806	0.99 (0.44–2.20)	0.976
*BMI, kg/m*^*2*^	1.01 (0.95–1.07)	0.758	1.03 (0.97–1.09)	0.378
< 30	1		1	
30–35	0.46 (0.18–1.19)	0.108	1.69 (0.70–4.08)	0.243
> 35	0.66 (0.29–1.51)	0.325	1.34 (0.52–3.43)	0.548
sBP at baseline	1.00 (0.98–1.02)	0.998	1.00 (0.98–1.02)	0.817
dBP at baseline	0.96 (0.93–1.00)	0.046	0.98 (0.94–1.02)	0.264
FBG, mg/dl	1.00 (1.00–1.01)	0.344	1.00 (0.99–1.01)	0.915
HbA1c, %	1.08 (0.87–1.35)	0.471	1.04 (0.81–1.35)	0.739
HbA1c < 7%	0.32 (0.08–1.35)	0.121	0.66 (0.20–2.22)	0.507
Total cholesterol, mg/dl	1.01 (1.00–1.01)	0.168	1.01 (1.00–1.02)	0.035
HDL, mg/dl	1.00 (0.97–1.04)	0.813	1.00 (0.97–10.04)	0.831
Triglycerides, mg/dl	1.001 (0.998–1.004)	0.539	1.003 (0.999–1.008)	0.174
LDLc, mg/dl	1.01 (1.00–1.02)	0.172	1.01 (1.00–1.02)^a^	0.056
eGFR, ml/min/1.73 m^2^	0.97 (0.96–0.99)	< 0.001	0.98 (0.96–1.00)	0.022
Diabetes duration, years	1.07 (1.03–1.10)	< 0.001	1.02 (0.99–1.06)	0.197
Diabetes duration > 10 years	2.13 (1.08–4.19)	0.029	1.51 (0.72–3.18)	0.278
> 50% coronary, carotid, or lower extremity artery stenosis	1.96 (0.68–5.63)	0.213	4.00 (1.97–8.14)	< 0.001
Diabetic kidney disease	2.17 (1.10–4.31)	0.026	1.91 (0.94–3.92)	0.076
*Anti-diabetic medications taken with GLP-1 RAs*				
Metformin + GLP-1	0.49 (0.17–1.41)	0.188	0.53 (0.20–1.39)	0.196
Sulphonylureas + GLP-1	2.01 (0.97–4.17)	0.062	1.49 (0.72–3.08)	0.282
Pioglitazone + GLP-1	0.35 (0.12–1.01)	0.052	0.94 (0.32–2.74)	0.909
Acarbose + GLP-1	3.72 (0.87–15.83)	0.076	NC	
Insulin + GLP-1	1.05 (0.50–2.19)	0.906	1.40 (0.68–2.90)	0.363
SGLT2i + GLP-1	NC		NC	
*Cardioactive therapies at baseline*				
Statins	0.70 (0.35–1.39)	0.306		
Ezetemibe	2.48 (0.75–8.17)	0.134		
Antiplatelet agents	0.90 (0.42–1.93)	0.783		
Anti-hypertensives	1.89 (0.73–4.90)	0.190		
ACEi o ARBs o MRAs	1.09 (0.53–2.24)	0.811		
*Other CV risk factors*				
Arterial hypertension	2.10 (0.74–5.97)	0.164		
Dyslipidemia	0.91 (0.45–1.82)	0.780		

NC, not calculated; ACEi, angiotensin-converting enzyme inhibitor; ARB, angiotensin II receptor blocker; MRA, mineralocorticoid receptor antagonist; CV, cardiovascular; LDL was calculated using the Friedewald formula; eGFR, estimated glomerular filtration rate calculated with the CKD-EPI formula

^a^Missing values in 27 subjects.

In secondary prevention, age (HR 1.05, 95% CI 1.00–1.11, *p* = 0.038), the presence of an arterial stenosis > 50% (HR 4.00, 95% CI 1.97–8.14, *p* < 0.001), total cholesterol (HR 1.01, 95% CI 1.00–1.02, *p* = 0.035), and kidney function (HR 0.98, 95% CI 0.96–1.00, *p* = 0.022) were associated to MACE risk (Table 6).

In the multivariable analysis, we considered the use of GPL-1 RA as a time dependent variable.

In the primary prevention group, change in BMI and glycated hemoglobin did not have an impact on MACE risk, while age at the time of GLP-1 initiation (HR 1.08, 95% CI 1.03–1.14, *p* = 0.001) and GLP-1 cessation by time (HR 3.40, 95% CI 1.82–6.32, *p* < 0.001) increased the risk of MACE, corrected by year of start of GLP-1 RA treatment. The impact of diabetes duration was not strictly significant, but again suggested an increase of MACE risk (HR 1.03 95% CI 1.00–1.07, *p* = 0.078).

Regarding the secondary prevention group, only GLP-1 RA cessation by time (HR 2.71, 95% CI 1.46–5.01, *p* = 0.002) increased the risk of MACE, corrected by year of start of GLP-1 RA treatment, while age at the time of GLP-1 RA initiation wasn’t strictly significant anymore, but still suggested an increased risk (HR 1.05, 95% CI 1.00–1.10, *p* = 0.067). Data are shown in Table 7.

**Table 7:** Results of multivariable time dependent Cox regression analysis for MACE

	HR (95% CI)	*p*
*Primary prevention*		
Age at beginning of GLP1, years	1.08 (1.03–1.14)	0.001
GLP-1 RA withdrawal by time	3.40 (1.82–6.32)	< 0.001
*Secondary prevention*		
GLP-1 RA withdrawal by time	2.71 (1.46–5.01)	0.002

Results are adjusted by year.

### Glycemic control and weight changes

Baseline characteristics (sex, age, diabetes duration) and weight changes, fasting blood glucose, and HbA1c during follow-up in primary and secondary prevention are shown in Table 8, comparing subjects who discontinued or not GLP-1 RA. No significant differences were observed in sex and age both in primary and secondary prevention group, diabetes duration was significantly longer in the primary prevention group who discontinued the GLP-RA treatment. There were significant differences in metabolic parameters between GLP-1 RA persistent and non-persistent subjects mainly in the primary prevention group.

**Table 8:** Weight changes, fasting blood glucose, and HbA1c during follow-up in primary and secondary prevention, comparing subjects who continued the GLP-1 RA to those who discontinued treatment

	Primary prevention					Secondary prevention				
	n	GLP-1 RA ongoing	n	GLP-1 RA discontinued	p	n	GLP-1 RA ongoing	n	GLP-1 RA discontinued	p
Sex (M)	236	121 (51.3%)	159	72 (45.3%)	0.243	92	75 (81.5%)	63	44 (69.8%)	0.091
Age at baseline	236	62.2 ± 8.9	159	60.7 ± 11.6	0.619	92	66.9 ± 7.1	63	65.5 ± 8.8	0.352
Diabetes duration at baseline	236	8.5 ± 6.6	159	10.4 ± 8.3	0.020	92	11.1 ± 6.5	63	13.4 ± 9.6	0.318
*Weight changes vs. baseline, kg*										
Year 1	226	− 2.93 ± 5.11	155	− 1.66 ± 6.01	0.021	92	− 2.33 ± 4.92	60	− 2.16 ± 4.90	0.983
Year 2	230	− 3.17 ± 6.12	151	− 1.42 ± 8.19	< 0.001	88	− 2.18 ± 6.05	58	− 2.07 ± 5.82	0.713
Year 4	132	− 4.14 ± 5.69	115	− 1.19 ± 8.26	< 0.001	40	− 4.46 ± 6.00	35	− 0.75 ± 6.84	0.006
Year 6	85	− 5.37 ± 7.14	88	− 2.15 ± 8.03	0.022	25	− 5.00 ± 8.58	18	− 2.53 ± 9.53	0.475
Year 8	47	− 6.64 ± 9.08	60	− 1.82 ± 11.79	0.010	15	− 8.71 ± 12.51	12	− 3.77 ± 14.86	0.463
Year 10	10	− 7.26 ± 11.10	16	− 2.46 ± 11.31	0.036	2	− 1.10 ± 1.13	2	− 19.10 ± 21.78	0.333
Final	235	− 2.75 ± 9.71	157	− 1.09 ± 9.77	0.002	91	− 2.69 ± 8.01	62	− 3.05 ± 8.99	0.677
95% CI final	235	− 4.00; − 1.50	157	− 2.63; 0.45		91	− 4.38; − 1.01	62	− 5.34;− 0.77	
*Fasting blood glucose, mg/dl*										
Baseline	231	169.7 ± 49.9	156	180.6 ± 58.6	0.052	89	158.6 ± 45.4	61	166.9 ± 56.6	0.331
Year 1	219	136.5 ± 31.6	150	156.5 ± 49.7	< 0.001	89	133.5 ± 34.0	57	150.9 ± 39.3	0.001
Year 2	225	137.4 ± 31.0	149	167.2 ± 60.2	< 0.001	89	131.6 ± 27.7	59	156.7 ± 54.8	0.002
Year 4	132	136.9 ± 32.0	115	164.1 ± 60.1	< 0.001	39	146.4 ± 37.7	33	152.1 ± 58.4	0.734
Year 6	82	141.6 ± 37.1	86	156.0 ± 54.7	0.114	24	140.4 ± 41.8	18	148.9 ± 47.6	0.806
Year 8	43	135.3 ± 40.7	59	145.6 ± 40.9	0.041	15	136.4 ± 45.5	13	156.0 ± 66.5	0.578
Year 10	9	121.8 ± 27.9	16	148.3 ± 83.6	0.607	2	123.0 ± 17.0	2	173.0 ± 33.9	0.333
Final	229	141.1 ± 37.0	152	155.8 ± 57.5	0.004	89	135.8 ± 33.6	61	154.2 ± 61.7	0.104
Baseline vs. final		< 0.001		< 0.001			0.001		0.046	
*HbA1c, %*										
Baseline	234	8.09 ± 1.22	154	8.41 ± 1.52	0.037	87	7.62 ± 1.11	63	8.23 ± 1.54	0.002
Year 1	223	6.90 ± 0.82	152	7.58 ± 1.30	< 0.001	89	6.86 ± 0.87	58	7.73 ± 1.02	< 0.001
Year 2	227	6.96 ± 0.94	148	7.77 ± 1.62	< 0.001	90	6.84 ± 0.74	59	7,83 ± 1.39	< 0.001
Year 4	133	6.88 ± 0.79	114	8.01 ± 1.58	< 0.001	38	7.28 ± 0.95	31	7.96 ± 1.33	0.014
Year 6	84	7.06 ± 1.07	87	7.80 ± 1.39	< 0.001	24	7.21 ± 1.10	18	7.81 ± 1.35	0.195
Year 8	46	6.75 ± 0.75	60	7.88 ± 1.81	< 0.001	15	6.80 ± 1.07	12	7.79 ± 1.04	0.004
Year 10	10	6.54 ± 0.53	17	7.83 ± 1.54	0.010	2	6.15 ± 0.35	2	8.50 ± 0.28	0.333
Final	233	7.08 ± 1.08	151	7.76 ± 1.46	< 0.001	91	6.85 ± 0.82	60	7.55 ± 1.40	0.002
Baseline vs. final		< 0.001		< 0.001			< 0.001		0.001	

"Final" refers to the last visit available for each patient

There were five severe hypoglycemic events, defined as hypoglycemia requiring external assistance for reversal, in four patients (0.7%).

## Discussion

In this real-life study of type 2 diabetes patients initiating a GLP-1 RA, longer duration of GLP-1 RA treatment was associated to a lower rate of a composite of non-fatal myocardial infarction or unstable angina, non-fatal stroke, and all-cause death in both primary and secondary prevention. To date, several metanalyses of RCTs have consistently shown the cardiovascular benefit of GLP-1 RAs, and growing evidence from real-world studies is supporting the efficacy of these drugs also in broader populations and in less controlled environments than those of RCTs [12–15, 17–20]. In fact, recent real-world studies comparing the cardiovascular effects of GLP-1 RAs versus other glucose-lowering drugs have shown a significant reduction in the composite cardiovascular outcome, to an extent varying from 30 to 33% [20, 21].

To date, the most convincing evidence of GLP-1 RA CV protection was obtained in secondary prevention populations, as CVOTs were initially designed as safety trials, and therefore enrolling subjects at very high CV risk was a strategy to increase the number of events and thus decrease trial duration and sample size. Specifically, the proportion of subjects in secondary prevention went from 70% in EXCSEL to 80% in SUSTAIN-6, and 100% in ELIXA and Harmony. The REWIND trial (dulaglutide vs. placebo), which included 69% of individuals in primary prevention, was an exception, and was the first study to suggest GLP-1 CV benefits even in subjects without a history of CV events. In fact, subgroup analyses from REWIND showed that MACE reduction in those treated with dulaglutide was similar in those with and without a history of previous CV events, but slightly missed statistical significance in both groups.

Similar to REWIND, the majority of the subjects included in our population was in primary CV prevention (72%), and the protection offered by continued GLP-1 RA treatment was similar in those with and without previous CV events. Also, GLP-1 RA treatment withdrawal (i.e.: before MACE or end of follow-up), considered as a time-dependent variable, significantly increased the risk of MACE in both groups. Interestingly, two of the most recent meta-analyses that included subgroup analyses of subjects with or without established CVD, suggest similar favorable effects in patients with and without CVD, but with a smaller reduction in absolute risk in those in primary prevention [12, 14].

Over half of the patients in the cohort were able to continue a GLP-1 RA in the long term (four to ten years). These subjects exhibited long-term benefits in terms of glycemic control, with a significant decrease in HbA1c of approximately -1% that was maintained over time, and a significant weight loss which increased in magnitude over the years, reaching a maximum of − 6.6 kg at eight years of follow-up.

When analyzing the possible variables associated with cardiovascular outcomes, we found that discontinuation of GLP-1 RA treatment was independently associated to a strong significant increase in the risk of developing a MACE. This finding is consistent with and reinforces the recent hypothesis that GLP-1 RAs appear to exert CV protection as long as patients are exposed to the individual drug for a sufficiently long amount of time. Indeed, the percentage of time of exposure to the investigational GLP-1 RA in individual CVOTs showed a positive correlation with MACE absolute risk reduction [22].

We may also note that our patients showed a greater treatment persistence compared to other real-world studies from other countries. In a retrospective study from a large cohort (8698 patients) in the United States, over half of the patients initiating GLP-1 RA were non-adherent and the majority (70.1%) discontinued therapy by 24 months [23]. Similarly, in another real-world study conducted in the UK among 589 patients initiating a GLP-1 RA, 45.2% and 64.7% of the subjects discontinued the treatment, at 12 and 24 months respectively [24].

Further, most of the patients during the observation period were treated with liraglutide (65.1% in primary prevention and 60.6% in secondary prevention), dulaglutide (46.1% in primary prevention and 51.6% in secondary prevention) and semaglutide (15.9% in primary prevention and 4.5% in secondary prevention), with liraglutide being prescribed more during the first years of observation and then partially switched to the newer weekly formulations. Indeed, all these molecules in their CVOTs have shown a superiority versus placebo in reducing MACE [6, 7, 9]. We may infer that the nature of the GLP-1 RAs used (long-acting and with easy-to-use devices) and the persistence to treatment observed in our cohort may have favored an exposure time sufficient to induce the clinical CV benefit, thus corroborating the link between treatment persistence and MACE HR.

Concerning the withdrawal of treatment, 17% of the patients (n = 96) experienced adverse events that led to drug discontinuation, in the vast majority being gastrointestinal symptoms (13%), a proportion slightly higher than in most CVOTs, in which drug discontinuation due to adverse events varied from 4.5% for once-weekly exenatide in EXSCEL (only gastrointestinal side effects were assessed) to 13.2% for subcutaneous semaglutide in SUSTAIN-6 [7, 8]. In 18% of the subjects, the molecule was withdrawn because glycemic or weight outcomes were not met, and therefore they were switched to a different class of diabetes medication. In this respect, in Italy, by regulatory decision, GLP-1 RAs were not allowed in combination with insulin until 2018, or with SGLT2-inhibitors until 2020. Therefore, patients needing a more intense treatment regimen had to be taken off GLP-1 RA. Indeed, 80% of those who withdrew the GLP-1 RA for inefficacy used insulin; 59% started a SGLT2i. Some concern may arise about the possible favorable interference deriving from SGLT-2i utilization, which have a proven CV protection [25], after GLP-1 RAs discontinuation. Indeed, if GLP-1 RA withdrawal by time is associated to an increased HR for MACE, the subsequent use of a SGLT2i could have partly mitigated (by adding CV protection) the effect of the sole GLP-1 RA discontinuation. In this view, we may speculate that if the patients who have discontinued the GLP-1 RA would have not subsequently started a SGLT-2i, they would have had an even greater risk of MACE.

Multiple mechanisms have been studied to explain the cardiovascular benefits of GLP-1 RAs. On one hand, GLP-1 RAs have been shown to effectively improve CV risk factors, including body weight, systolic blood pressure, LDL cholesterol and triglyceride concentrations, and glycemic control [16]. On the other hand, GLP-1 RAs also seem to directly interfere with atherogenic processes. Animal studies and studies on human cells indicate that GLP-1 RAs interfere with the atherogenesis process through GLP-1 receptors expressed by various cell populations involved in plaque development and rupture, including endothelial cells, monocytes, macrophages, and vascular smooth muscle cells [26–34]. The overall result is slower plaque progression and plaque stabilization. Epigenetic mechanisms may play a relevant role, since GLP-1 RAs may revert the DNA hypomethylation induced by hyperglycemia, once again leading to decreased activation of pro-inflammatory and pro-atherogenic pathways [35]. These mechanisms, involving anti-inflammatory and anti-atherogenic effects which lead to a plaque stabilization over time, may potentially explain the time-dependent CV risk reduction observed in our cohort. Remarkably, in our study population, longer duration of GLP-1 RA treatment positively affected metabolic outcomes in the primary CV prevention group, in accord with similar evidence reporting that GLP-1 treatment was statistically associated with a long-lasting decrease in HbA1c over time [36]. Indeed, tighter glycemic control alone could counteract the development of atherosclerosis in its early stages, but not in the presence of overt vascular damage [37] and intensive glucose control has been shown to reduce the risk of major CV events, mainly in type 2 diabetes patients without evidence of macrovascular disease [38]. Interestingly, an association between the magnitude of HbA1c reduction (versus placebo) and MACE hazard ratio in an analysis of GLP-1 RA CVOTs was previously suggested [39]. Also, a mediation analysis of the REWIND trial concluded that the improvement in HbA1c was a potential mediator of CV protection, responsible for up to 82% of the total effect on MACE [40].

In fact, in our study, we observed a sustained reduction of HbA1c in patients treated with GLP-1 RA, with most values during follow up being under 7%. This could be considered a remarkable result in a real-life setting, in which the treatment targets have changed over the years according to guidelines’ updates.

We are aware that the lack of a control group prevents further speculations, but we may note that in our population the incidence of MACE was 26 per 1000 person-years, which is relatively close to the rate observed in the dulaglutide group of the REWIND trial (namely 35.8 per 1000 person-years), further corroborating the expected beneficial CV effect of GLP-1 RAs use [41].

This study has some limitations. The first is its retrospective nature which may carry the risk of measurements errors, non-standardized data elements and selection bias. Another limitation is the lack of a control group; to overcome this issue we applied a propensity score matching with the rest of diabetic population attending our center (i.e.: subjects treated with SGLT2 inhibitors or with DPP4 inhibitors or with insulin and never prescribed GLP-1 RAs), but the number yielded was too low to obtain reliable statistical results. Treatment adherence was self-reported by patients, which may raise concerns due to its vulnerability to social desirability and memory biases that tend to overestimate the degree to which patients execute medication regimens. Also, patients were followed at a single tertiary university center, thus involving a population that may be different to other territorial realities. These aspects may limit the generalizability of the conclusion drawn here.

Strengths of the present study include the relatively long follow-up period, compared to most CVOTs and real-world studies, and the real-world population of subjects, most of whom without established CVD. Additionally, at the time most subjects entered the study, the results from CVOTs were not yet available, and thus did not heavily affect the selection of patients based on CV risk parameters, but rather more based on metabolic parameters, such as HbA1c not at target, or need for weight loss.

## Conclusions

Our study showed that in a real-life population of type 2 diabetes patients, GLP-1 RAs may reduce the risk of MACE in a time-dependent manner, thus establishing an association between duration of treatment with GLP-1 RAs and CV outcomes. Since discontinuation of GLP-1 RAs increased the risk of MACE this treatment should be maintained over time, whenever possible.

Further investigations involving long term treatment with GLP-1 RA on larger real-world populations are warranted to confirm the time-dependent benefit on CV outcomes.

## Data Availability

The dataset supporting the conclusions of this article is available in the Zenodo repository, https://doi.org/10.5281/zenodo.7533472.

## Abbreviations

ACE: Angiotensin-converting enzyme
ARB: Angiotensin receptor blockers
BMI: Body mass index
BP: Blood pressure
CI: Confidence interval
CV: Cardiovascular
CVD: Cardiovascular disease
CVOT: Cardiovascular outcome trial
DPP4: Dipeptidyl peptidase 4
FBG: Fasting blood glucose
GLP-1 RA: Glucagon-like peptide-1 receptor agonist
HF: Heart failure
HR: Hazard ratio
LADA: Latent autoimmune diabetes of the adult
MACE: Major adverse cardiovascular events
MI: Myocardial infarction
MRA: Mineralocorticoid receptor antagonists
PCSK-9: Proprotein convertase subtilisin/kexin type 9
RCT: Randomized controlled trial
SGLT2: Sodium-glucose cotransporter 2

## References

[1] Nauck MA, Heimesaat MM, Orskov C, Holst JJ, Ebert R, Creutzfeldt W (1993). Preserved incretin activity of glucagon-like peptide 1 [7-36 amide] but not of synthetic human gastric inhibitory polypeptide in patients with type-2 diabetes mellitus. J Clin Invest.

[2] Nauck MA, Kleine N, Ørskov C, Holst JJ, Willms B, Creutzfeldt W. Normalization of fasting hyperglycaemia by exogenous glucagon-like peptide 1 (7–36 amide) in type 2 (non-insulin-dependent) diabetic patients. Diabetologia [Internet]. 1993 [cited 2022 Oct 1];36(8):741–4. Available from: https://pubmed.ncbi.nlm.nih.gov/8405741/10.1007/BF00401145 8405741

[3] Nauck MA, Heimesaat MM, Behle K, Holst JJ, Nauck MS, Ritzel R (2002). Effects of glucagon-like peptide 1 on counterregulatory hormone responses, cognitive functions, and insulin secretion during hyperinsulinemic, stepped hypoglycemic clamp experiments in healthy volunteers. J Clin Endocrinol Metab.

[4] Wettergren A, Schjoldager B, Mortensen PE, Myhre J, Christiansen J, Holst JJ (1993). Truncated GLP-1 (proglucagon 78–107-amide) inhibits gastric and pancreatic functions in man. Dig Dis Sci.

[5] Pfeffer MA, Claggett B, Diaz R, Dickstein K, Gerstein HC, Køber LV, et al. Lixisenatide in patients with type 2 diabetes and acute coronary syndrome. N Engl J Med. 2015;373(23):2247–57. 10.1056/NEJMoa1509225 26630143

[6] Marso SP, Daniels GH, Brown-Frandsen K, Kristensen P, Mann JFE, Nauck MA (2016). Liraglutide and cardiovascular outcomes in type 2 diabetes. N Engl J Med.

[7] Marso SP, Bain SC, Consoli A, Eliaschewitz FG, Jódar E, Leiter LA (2016). Semaglutide and cardiovascular outcomes in patients with type 2 diabetes. N Engl J Med.

[8] Holman RR, Bethel MA, Mentz RJ, Thompson VP, Lokhnygina Y, Buse JB (2017). Effects of once-weekly exenatide on cardiovascular outcomes in type 2 diabetes. N Engl J Med.

[9] Gerstein HC, Colhoun HM, Dagenais GR, Diaz R, Lakshmanan M, Pais P (2019). Dulaglutide and cardiovascular outcomes in type 2 diabetes (REWIND): a double-blind, randomised placebo-controlled trial. The Lancet.

[10] Hernandez AF, Green JB, Janmohamed S, D’Agostino RB, Granger CB, Jones NP (2018). Albiglutide and cardiovascular outcomes in patients with type 2 diabetes and cardiovascular disease (Harmony Outcomes): a double-blind, randomised placebo-controlled trial. The Lancet.

[11] Husain M, Birkenfeld AL, Donsmark M, Dungan K, Eliaschewitz FG, Franco DR (2019). Oral semaglutide and cardiovascular outcomes in patients with type 2 diabetes. N Engl J Med.

[12] Kristensen SL, Rørth R, Jhund PS, Docherty KF, Sattar N, Preiss D (2019). Cardiovascular, mortality, and kidney outcomes with GLP-1 receptor agonists in patients with type 2 diabetes: a systematic review and meta-analysis of cardiovascular outcome trials. Lancet Diabetes Endocrinol.

[13] Bethel MA, Patel RA, Merrill P, Lokhnygina Y, Buse JB, Mentz RJ (2018). Cardiovascular outcomes with glucagon-like peptide-1 receptor agonists in patients with type 2 diabetes: a meta-analysis. Lancet Diabetes Endocrinol.

[14] Marsico F, Paolillo S, Gargiulo P, Bruzzese D, Dell’Aversana S, Esposito I (2020). Effects of glucagon-like peptide-1 receptor agonists on major cardiovascular events in patients with Type 2 diabetes mellitus with or without established cardiovascular disease: a meta-analysis of randomized controlled trials. Eur Heart J.

[15] Giugliano D, Scappaticcio L, Longo M, Caruso P, Maiorino MI, Bellastella G (2021). GLP-1 receptor agonists and cardiorenal outcomes in type 2 diabetes: an updated meta-analysis of eight CVOTs. Cardiovasc Diabetol..

[16] Nauck MA, Meier JJ, Cavender MA, el Aziz MA, Drucker DJ (2017). Cardiovascular actions and clinical outcomes with glucagon-like peptide-1 receptor agonists and dipeptidyl peptidase-4 inhibitors. Circulation.

[17] Zelniker TA, Wiviott SD, Raz I, Im K, Goodrich EL, Furtado RHM (2019). Comparison of the effects of glucagon-like peptide receptor agonists and sodium-glucose cotransporter 2 inhibitors for prevention of major adverse cardiovascular and renal outcomes in type 2 diabetes mellitus: systematic review and meta-analysis of cardio. Circulation.

[18] Bellastella G, Maiorino MI, Longo M, Scappaticcio L, Chiodini P, Esposito K, et al. Glucagon-like peptide-1 receptor agonists and prevention of stroke systematic review of cardiovascular outcome trials with meta-analysis. Stroke. 2020;666–9. 10.1161/STROKEAHA.119.027557 31813360

[19] Mannucci E, Dicembrini I, Nreu B, Monami M (2020). Glucagon-like peptide-1 receptor agonists and cardiovascular outcomes in patients with and without prior cardiovascular events: an updated meta-analysis and subgroup analysis of randomized controlled trials. Diabetes Obesity and Metabolism.

[20] Caruso I, Cignarelli A, Sorice GP, Natalicchio A, Perrini S, Laviola L, et al. Cardiovascular and renal effectiveness of GLP-1 receptor agonists vs. other glucose-lowering drugs in type 2 diabetes: a systematic review and meta-analysis of real-world studies. Metabolites. 2022;12(2). 10.3390/metabo12020183 PMC8879165 35208256

[21] Longato E, di Camillo B, Sparacino G, Tramontan L, Avogaro A, Fadini GP (2021). Cardiovascular outcomes after initiating GLP-1 receptor agonist or basal insulin for the routine treatment of type 2 diabetes: a region-wide retrospective study. Cardiovasc Diabetol..

[22] Caruso I, Cignarelli A, Laviola L, Giorgino F (2022). GLP-1 receptor agonists for cardiovascular protection: a matter of time. Diabetes Care..

[23] Weiss T, Carr RD, Pal S, Yang L, Sawhney B, Boggs R (2020). Real-world adherence and discontinuation of glucagon-like peptide-1 receptor agonists therapy in type 2 diabetes mellitus patients in the United States. Patient Prefer Adherence.

[24] Weiss T, Yang L, Carr RD, Pal S, Sawhney B, Boggs R, et al. Real-world weight change, adherence, and discontinuation among patients with type 2 diabetes initiating glucagon-like peptide-1 receptor agonists in the UK. BMJ Open Diabetes Res Care. 2022;10(1). 10.1136/bmjdrc-2021-002517 PMC8804648 35101924

[25] Braunwald E (2022). Gliflozins in the management of cardiovascular disease. N Engl J Med.

[26] Bray JJH, Foster-Davies H, Salem A, Hoole AL, Obaid DR, Halcox JPJ (2021). Glucagon-like peptide-1 receptor agonists improve biomarkers of inflammation and oxidative stress: a systematic review and meta-analysis of randomised controlled trials. Diabetes Obes Metab.

[27] Koshibu M, Mori Y, Saito T, Kushima H, Hiromura M, Terasaki M (2019). Antiatherogenic effects of liraglutide in hyperglycemic apolipoprotein E-null mice via AMP-activated protein kinase-independent mechanisms. Am J Physiol Endocrinol Metab..

[28] Rakipovski G, Rolin B, Nøhr J, Klewe I, Frederiksen KS, Augustin R (2018). The GLP-1 Analogs liraglutide and semaglutide reduce atherosclerosis in ApoE −/− and LDLr −/− mice by a mechanism that includes inflammatory pathways. JACC Basic Transl Sci.

[29] Marquis-Gravel G, Tardif JC (2021). Glucagon-like peptide 1 receptor agonists, carotid atherosclerosis, and cardiovascular outcomes. Diabetes Care..

[30] Bruen R, Curley S, Kajani S, Lynch G, O’Reilly ME, Dillon ET (2019). Liraglutide attenuates preestablished atherosclerosis in apolipoprotein E-deficient mice via regulation of immune cell phenotypes and proinflammatory mediators. J Pharmacol Exp Therap..

[31] Nikolic D, Giglio RV, Rizvi AA, Patti AM, Montalto G, Maranta F (2021). Liraglutide reduces carotid intima-media thickness by reducing small dense low-density lipoproteins in a real-world setting of patients with type 2 diabetes: a novel anti-atherogenic effect. Diabetes Therapy.

[32] Rizzo M, Rizvi AA, Patti AM, Nikolic D, Giglio RV, Castellino G (2016). Liraglutide improves metabolic parameters and carotid intima-media thickness in diabetic patients with the metabolic syndrome: an 18-month prospective study. Cardiovasc Diabetol..

[33] Bruen R, Curley S, Kajani S, Crean D, O’Reilly ME, Lucitt MB (2017). Liraglutide dictates macrophage phenotype in apolipoprotein E null mice during early atherosclerosis. Cardiovasc Diabetol..

[34] Chang W, Zhu F, Zheng H, Zhou Z, Miao P, Zhao L (2019). Glucagon-like peptide-1 receptor agonist dulaglutide prevents ox-LDL-induced adhesion of monocytes to human endothelial cells: An implication in the treatment of atherosclerosis. Mol Immunol.

[35] Scisciola L, Rizzo MR, Cataldo V, Fontanella RA, Balestrieri ML, D’Onofrio N (2020). Incretin drugs effect on epigenetic machinery: New potential therapeutic implications in preventing vascular diabetic complications. FASEB J.

[36] Kwan AYM, Gerstein HC, Basile J, Xavier D, Maldonado JM, Raha S, et al. HbA 1c reduction in dulaglutide-treated patients irrespective of duration of diabetes, microvascular disease, and BMI: a post hoc analysis from the REWIND trial. 2022. 10.2337/dc21-1160 10.2337/dc21-1160 PMC8918189 35043140

[37] Laiteerapong N, Ham SA, Gao Y, Moffet HH, Liu JY, Huang ES (2019). The legacy effect in type 2 diabetes: impact of early glycemic control on future complications (The Diabetes & Aging Study). Diabetes Care..

[38] Turnbull FM, Abraira C, Anderson RJ, Byington RP, Chalmers JP, Duckworth WC (2009). Intensive glucose control and macrovascular outcomes in type 2 diabetes. Diabetologia.

[39] Caruso I, Cignarelli A, Natalicchio A, Perrini S, Laviola L, Giorgino F (2020). Commentary: Glucose control: not just a bystander in GLP-1RA-mediated cardiovascular protection. Metabolism..

[40] Konig M, Riddle MC, Colhoun HM, Branch KR, Atisso CM, Lakshmanan MC, et al. Exploring potential mediators of the cardiovascular benefit of dulaglutide in type 2 diabetes patients in REWIND. 2021. 10.1186/s12933-021-01386-4 10.1186/s12933-021-01386-4 PMC8466679 34563178

[41] Dagenais GR, Rydén L, Leiter LA, Lakshmanan M, Dyal L, Probstfield JL (2020). Total cardiovascular or fatal events in people with type 2 diabetes and cardiovascular risk factors treated with dulaglutide in the REWIND trail: a post hoc analysis. Cardiovasc Diabetol..

